# Obesity and Healthcare Avoidance: A Systematic Review

**DOI:** 10.3934/publichealth.2015.1.56

**Published:** 2015-03-11

**Authors:** Robert D McGuigan, Jenny M Wilkinson

**Affiliations:** 1 South West Pathology Service, Albury, NSW, 2640, Australia; 2 School of Biomedical Sciences, Charles Sturt University, Wagga Wagga, NSW, 2678, Australia

**Keywords:** obesity, healthcare avoidance, preventative health

## Abstract

This review addresses the issue of health care avoidance and obesity. English language journal articles published between 1990 and 2012 that addressed the review question; “is being overweight or obese an unrecognized factor in healthcare avoidance?” were located using major databases. A modified JADAD scoring system was then used to assess papers. Ten papers were identified which directly addressed the review question. A positive relationship exists between obesity and healthcare avoidance. The major contributory factors were being female, have a diagnosed mental health problem and perceived or actual bias and discrimination by health professionals. The review also highlights the importance of the relationship between healthcare professionals and their patients, and the physical environment in which interactions occur as these may contribute to avoidance behaviors. Concern about obesity is rising and while there has been much discussion about strategies to reduce obesity this review highlights the need for thinking more broadly about the way in which overweight and obese individuals interact with preventative health strategies.

## 1. Introduction

Obesity is the most common metabolic condition in industrialized nations. International estimates of the condition vary widely and may depend on cultural and socioeconomic factors in addition to the age group, timeframe studied, and the definition of obesity used [1]. The last study that attempted to estimate the cost of obesity in some western health budgets was nearly a decade ago and even at that time it was a significant health budget expenditure item in industrialized countries; Canada 2.4% of the health budget, U.S.A. 5.5–6.8%, Australia 2%, New Zealand 2.5%, Netherlands 4% and France 2% [1]. From a medical stand point, obesity is usually quantified using body mass index (BMI; kg/m^2^). Individuals with a BMI in the range 25–30 kg/m^2^ are considered overweight, 30–35 kg/m^2^ are considered obese and greater than 35 kg/m^2^ are considered seriously obese [2]. Worldwide comparisons of obesity rates is however difficult as different definitions of obesity are used to categorize the severely obese in some countries (i.e., from 25 to 30 kg/m^2^) and it is this category that was used to obtain health budget expenditures [1]. At a population level there are many factors that contribute to this apparent increase in obesity, for example increasing worldwide food production, increasing mechanization of food production, lifestyles that require less physical activity and the change from high fiber, low fat and low carbohydrate foods to higher fat and carbohydrate content foods [2].

The biological determinants of obesity are incompletely understood however the familial nature of obesity in some suggests it is a polygenic condition [2]. The psychological influences on the development of obesity are many and varied and may be related to gender issues [3]–[7], cultural differences [2], inappropriate childhood learned behaviors centering around food intake [8] or inappropriate reward strategies involving food intake by the individuals themselves and those around them [5],[9]. There are also specific mental, physical and biological problems associated with obesity [2]. Being overweight or obese is recognized by health professionals as being directly proportional to the health risk to numerous organ systems [10]. The percentages of overweight and obese individuals are also more prevalent in populations who smoke or have excessive alcohol intake and this usually equates to lower educational levels and household incomes [11]. Premature death due to obesity-related organ damage is responsible for 9.5% of all life years lost relative to life expectancy for females and 8.4% for males [12]. Preventative screening for such things as cancer, high blood pressure and lipid profiles reduces premature death in both normal weight and overweight or obese individuals. However, the overweight and obese are especially in need of this preventative screening because of their poorer health [11].

As being overweight or obese is becoming an increasing problem worldwide and given that being overweight or obese is a direct cause of poor health and premature death preventative health screening programs should be designed so as to be inclusive as possible of this group of individuals. Normal weight range individuals perceive how others see them and how they see themselves differently to how an overweight or obese person sees the world around them and themselves and this review seeks to see if this has an effect on health-seeking behavior [13]. Any designing of preventative screening programs should specifically take into account this difference in attitude and resulting behaviors. As health professionals do this testing they may need to refine how they treat this group of individuals so as to minimize adverse health effects of being overweight or obese, especially in the area of preventative screening technologies.

## 2. Materials and Method

The systematic review question was; is being overweight or obese an unrecognized factor in healthcare avoidance? A modified JADAD systematic review question regime [14] was applied to journal articles as follows:

- Q1 Is there a description of how the sample population was selected (sample population inclusion and exclusion criteria)?
- Q2 Is the sample size adequately described?
- Q3 Does the paper have its methodology described?
- Q4 Does the paper address the systematic review question?

Articles for consideration in the review were located by searching the following databases; PubMed, Google scholar; CINAHL, OVID and MEDLINE. Papers published between January 1^st^ 1990 and 31^st^ of December 2011 were used so as to give enough articles and papers which met the inclusion and exclusion criteria while still reflecting contemporary attitudes and behaviours. Several keyword combinations were used in the search engines; “obesity, testing, unrecognized factor,” were used initially. The phrase “body mass index/BMI” was substituted for obesity to further refine the search. Another article search was used in the same databases using the words “healthcare avoidance” and “obesity.” A final search was performed using the words “non-compliance with screening” and “BMI.” The website of the journal Obesity was also searched for relevant articles. The reference lists of papers that appeared to address the review question were also searched for additional papers which met the inclusion/exclusion criteria.

An initial cull of articles and papers that appeared to address the review question was carried out. Fifty three papers that appeared to meet the inclusion criteria were selected from this initial cull and were subjected to further analysis. From this further analysis twenty eight papers were selected that appeared to address some aspect of the review question; of these ten papers described research studies which directly addressed the review question. These were assessed independently by both authors and a score from one to five assigned for each of the modified JADAD questions (1 = not described, 2 = poorly described, 3 = adequately described, 4 = well described, 5 = comprehensively described). A combined consensus score was then arrived at by both assessors and this score determined the articles and papers to be excluded and included in the review process.

## 3. Results and Discussion

From the literature a number of issues were identified which contribute to healthcare avoidance by those who are overweight or obese. These issues focused on gender, psychological well-being and actual or perceived bias. This review has also identified that there is a lack of original research on the topic of healthcare avoidance in the overweight and obese. Further, many of the identified papers had significant flaws and scored poorly in terms of their methodological quality (Table 1). In addition literature on this topic was found to focus primarily on women's healthcare issues [15],[16]. A positive relationship exists between the delay or avoidance of healthcare and excessive BMI. The major causes of healthcare avoidance in the obese and overweight identified in the literature examined are gender-based [15], psychological [16] and bias issues [17]. Overweight and obese individuals because of generalized poor health, also have greater contact with their physicians [18]. However this does not correlate to increased rates of preventative screening in this group, in fact the opposite is true. Physicians recommend preventative screening and other interventions to their patients whether they are obese or not and are just as likely to suggest obese persons present for testing as they are to normal BMI individuals [18]. The lower adherence to these recommendations by overweight or obese individuals to preventative screening and testing appears not to be due to the lack of physician recommendations but rather to lower adherence once the recommendation is given.

Men and women differ in levels of psychological well-being as a function of BMI [3],[4]. It is proposed that obese women are much more likely to internalize cultural norms that link thinness to physical attractiveness and thus view themselves more critically than men [4]. It therefore follows that even though both sexes will have lesser rates of adherence to physician recommendations than their normal BMI cohort obese and overweight women will present less often for screening intervention examinations especially colonoscopies [15],[16], breast mammograms and Pap smears [19]–[22]. Overweight and obese males by comparison were much more likely to comply with screening and other testing requests from their doctors and other health professionals [15]. Physicians may also more actively encourage overweight and obese males to participate in preventative screening than their female cohort, the reasoning for this is however unclear.

**Table 1.** Papers reviewed for inclusion in the systematic review.

Authors	Consensus scores Q1 to Q4	Comments
Drury & Louis (2002)	5,5,4,5	Female respondents (*n* = 216)
		A positive relationship was found between obesity and delay/avoidance of health care that was unrelated to satisfaction with actual healthcare provision. Actual or perceived disapproval from health care providers in relation to bodyweight was a contributing factor to healthcare avoidance.
Heo et al. (2004)	2,3,3,5	Male and female respondents aged over 50 (*n* = 84,284)
		Women with increased BMI had lower rates of sigmoidoscopy for colorectal cancer screening compared with those with normal BMI; in contrast males with higher BMI had higher rates of screening. Fecal occult blood test rates were not associated with BMI in either males or females.
Rosen & Schneider (2004)	4,4,4,4	Adults aged 51 to 80 years (*n* = 52,886)
		Morbidly obese women were less likely to have participated in screening for colorectal cancer than other weight groups or males or all weight groups.
Schwartz et al. (2003)	4,4,4,2	Researchers and clinicians attending an obesity conference; self-administered survey tool (*n* = 389)
		Health professionals, including those involved in clinical management of obesity, showed a strong pro-thin, anti-fat implicit bias. Views which reinforced negative stereotypes of overweight individuals were also endorsed by respondents.
Ferrante et al. (2008)	4,4,4,5	Female respondents only (*n* = 8,289)
		Despite equivalent rates of recommendation by physicians for mammography and Pap smears between obese and normal weight women, obese individuals were less likely to have adhered to these recommendations.
Coughlin et al. (2004)	4,5,4,4	Female respondents only. (*n* = 56,528)
		After adjusting for a range of factors associated with screening there was no difference between the percentage of obese and normal-weight women who had recent mammograms or Pap smears.
Ostbye et al. (2005)	4,5,4,4	Analysis of 2 datasets: HRS study, women 50–61 years (*n* = 4,439); AHEAD study, males and females, 70 years or more (*n* = 6,200)
		Higher BMI was associated with lower level of mammography, Pap smear screening in white middle-aged women and receipt of the influenza vaccine in the elderly.
Wee et al. (2000)	4,5,4,5	Female respondents aged 18 to 75 years with no hysterectomy (*n* = 8,394)
		Even after accounting for known barriers to breast and cervical cancer screening women who are overweight or obese are less likely to undergo mammography or Pap smear screening.
Wee et al. (2004)	4,4,4,5	Deals with less common obesity healthcare avoidance issues. Caucasian and con-Caucasian female respondents aged 50 to 70 years (*n* = 5,277)
		After accounting for confounders such as health access and illness higher BMI was associated with reduced likelihood of undergoing careening for breast cancer in white women.
Pirraglia et al. (2004)	5,5,4,2	Female respondents aged 42–52 years (*n* = 3,302)
		BMI greater than or equal to 30 kg/m^2^ have significant depressive illness which is a barrier to screening for cervix and breast cancer.

In contrast with the strong link between being overweight or obese and having poorer physical health, the link between excessive body weight and psychological well-being is less clear cut. In general the obese and overweight suffer from increased rates of psychological disorders including anxiety and depressive illnesses [3] and this has been identified in several studies as a factor leading to non-compliance with medical treatment and avoidance of preventative screening interventions [16],[23],[24]. Patients suffering from depressive illness are three times less likely to be compliant with medical treatment than their non-depressed counterpart [23]. It is also much more likely that obese and overweight women will suffer from these psychological disorders than men [4]. If a woman has a depressive disorder she is also more likely to suffer from social phobias than her overweight and obese male counterpart [3], this especially includes health care avoidance [18],[24].

Several authors have noted that overweight and obese individuals are targets of discrimination [13],[17]. Overweight and obese individuals are subjected to bias, inappropriate humor, stigmatization and ridicule by many levels of society. As this may include health professionals the perceived or actual bias by health professionals is a major concern when considering health screening. The relationship between obesity and higher medical costs is obviously influenced by the pathophysiology of obesity but may also result in a vicious cycle. Obese patients may be reluctant to seek medical help because of perceived or actual weight bias, thus increasing the likelihood of medical problems with their associated healthcare costs.

The literature examined supports a strong association between being overweight or obese and delay or avoidance of healthcare which is independent of the level of physician involvement or physician recommendations to this group of individuals. There is also strong evidence that obese or overweight women are more at risk from obesity-related diseases than their male counterpart because the overweight or obese woman is more susceptible to psychological abnormalities including social phobias and depressive illnesses. The social phobias and depressive illnesses can be further magnified by perceived or actual weight bias by their peers and health professionals that either interact with, or, treat this group of people. This causes isolation, stigmatization and low self-esteem. The possibility then exists for a weight bias vicious cycle to be created in the minds of the overweight or obese reinforcing further delaying or avoiding healthcare.

## 4. Conclusions

The overweight and obese in society are a vulnerable group both due to their increased chronic disease problems, delaying or avoiding healthcare and premature mortality. The simple act of a general practitioner taking blood pressure measurements, suggesting a lipid profile be performed or weighing a normal BMI patient is a relatively benign affair, this is not so for a patient with excessive BMI. These simple acts are something to be avoided or at the very least suffered as seldom as possible. The confounding issues of gender and mental health add to the healthcare problems of this group. The American National Taskforce for the prevention and treatment of obesity was advising as far back as 2002 that simple redesigning of the patient area of general practitioner's office, especially seating to accommodate the larger patient was thought to be advantageous, also, where and how patients are weighed were considered cost-effective steps to remedying the phenomenon of healthcare avoidance by the overweight and obese [10]. However a great deal more research needs to be done on this topic so as stratagems can be developed to better maintain the health of the overweight and obese in society.
